# Chronic kidney disease awareness, prevention and policy gaps with implications for the Nigerian Navy: a scoping review

**DOI:** 10.4314/ahs.v26i2.12

**Published:** 2026-06

**Authors:** Funke Sulyman, Rafiat Ajoke Anokwuru, Aliyu Danjuma, Odekunle Bola Odegbemi

**Affiliations:** 1 Nursing Science Department, Babcock University, Ilishan-Remo, Ogun State, Nigeria; 2 Nursing Science Department, Naval Medical Centre, Victoria Island, Lagos State, Nigeria; 3 Perioperative Nursing Department, Ahmadu Bello University Teaching Hospital, Zaria, Kaduna State, Nigeria; 4 Medical Laboratory Science Department, Edo State University, Uzairue, Edo State, Nigeria; 5 Medical Laboratory Science Department, Nigerian Navy Hospital, Warri, Delta State, Nigeria

**Keywords:** Chronic Kidney Disease, Nigerian Navy, Military Health System, Nephrology, Dialysis Access, Scoping Review, Non-Communicable Diseases, Occupational Risk

## Abstract

**Background:**

Chronic kidney disease (CKD) is a major global health concern affecting over 850 million people. Among military personnel, CKD poses unique operational and occupational challenges, yet information on awareness, prevention, and service delivery within the Nigerian Navy (NN) is limited.

**Objectives:**

To map existing evidence on CKD knowledge, prevention practices, healthcare services, and policy frameworks relevant to the NN, identify service and evidence gaps, and propose feasible interventions.

**Methods:**

A scoping review guided by the PRISMA-ScR framework was conducted. Literature published between 2000 and April 2024 was searched across PubMed, African Journals Online, and Google Scholar, complemented by grey literature from the Nigerian Navy Medical Services and the Ministry of Defence. Studies were selected using the Population–Concept–Context (PCC) framework and thematically synthesized across five domains: epidemiology, screening, workforce capacity, dialysis access, and governance.

**Results:**

Eight studies met the inclusion criteria. Evidence showed fragmented CKD prevention, low awareness, absence of a renal registry, gaps in workforce capacity, and restricted dialysis services confined to the Naval Medical Centre Victoria Island, and the Nigerian Navy Reference Hospital Ojo. No dedicated CKD or NCD policy exists.

**Conclusion:**

Strengthening screening, establishing a Navy CKD registry, and expanding tele-nephrology could enhance early detection, coordination, and operational readiness.

## Introduction

Chronic kidney disease (CKD) represents a major and escalating global public health challenge. Recent estimates indicate that over 850 million people worldwide live with kidney disease, corresponding to a global prevalence of 10–16% among adults^1^. The World Health Organization projects CKD to become the fifth leading cause of years of life lost by 2040, driven largely by the increasing prevalence of hypertension, diabetes mellitus, and obesity in low- and middle-income countries^2^,^3^. In sub-Saharan Africa, the burden of CKD is particularly high because of limited access to early screening, delayed diagnosis, and inadequate renal replacement therapy services^4^.

In Nigeria, hospital and community-based studies have reported CKD prevalence ranging from 11% to 26%, depending on diagnostic criteria and study population^5^,^6^. The disease disproportionately affects adults in their economically productive years, contributing to reduced quality of life and a heavy financial burden for patients and families. Despite these statistics, national data remain sparse, and renal disease surveillance systems are poorly developed. Nigeria lacks a centralized renal registry, and public awareness of CKD risk factors, such as uncontrolled hypertension, diabetes, and prolonged use of nephrotoxic agents, remains low^7^.

Military populations face distinctive occupational and environmental exposures that may predispose them to kidney dysfunction. These include chronic dehydration, recurrent heat stress, long deployments with limited medical access, high physical exertion, and occasional exposure to toxic agents such as fuels, solvents, and cleaning chemicals used aboard ships or in workshops^8^. Studies among military personnel in other countries have identified CKD as an emerging non-communicable disease of operational importance, with implications for medical deployment standards and force readiness^9^. The Nigerian Navy (NN), whose personnel serve in both maritime and terrestrial operations, may face similar or even higher risk, yet published data describing the pattern of renal disease, awareness, and preventive practices within this population are extremely limited.

Globally, several military health systems have recognized CKD as a critical component of occupational health and have implemented structured preventive programs. The United States Navy, for instance, integrates serum creatinine, estimated glomerular filtration rate (eGFR), and urinalysis into routine annual physical examinations, supported by a comprehensive renal registry managed under the Bureau of Medicine and Surgery^10^. The Indian Armed Forces Medical Services integrate CKD monitoring, tele-nephrology, and structured annual renal checks across their tri-service medical system. In contrast, the Nigerian Navy Medical Services (NNMS) have not yet institutionalized a formal CKD screening policy, renal registry, or structured health-education framework. Dialysis services remain limited to only two naval facilities, the Naval Medical Centre, Victoria Island, and the Nigerian Navy Reference Hospital, Ojo, reflecting significant infrastructural and human-resource constraints.

Mapping current knowledge, screening practices, and policy gaps is therefore essential for evidence-based improvement of renal health services within the Nigerian Navy. A scoping review approach guided by the PRISMA-ScR framework was adopted to synthesize available literature on CKD awareness, prevention, healthcare delivery, and governance, with specific implications for the Nigerian Navy. This review aims to identify knowledge deficits, service limitations, and opportunities for strengthening early detection, workforce capacity, and institutional readiness for CKD prevention and management in Nigeria's naval health system.

## Methods

This scoping review was conducted in accordance with the methodological framework proposed by Arksey and O'Malley, further refined by Levac et al., and reported following the PRISMA Extension for Scoping Reviews (PRISMA-ScR) guidelines^11^,^12^.

### Stage 1: Identifying the Research Question

The review addressed the following research question: What evidence exists on chronic kidney disease (CKD) awareness, screening, service availability, and policy frameworks relevant to the Nigerian Navy, and what gaps require urgent attention?

### Stage 2: Identifying Relevant Studies

A comprehensive literature search was conducted for publications between January 2000 and April 2024 across PubMed, African Journals Online (AJOL), and Google Scholar. Grey literature was additionally sourced from Nigerian Navy Medical Services (NNMS) reports and Ministry of Defence publications.

Search terms included combinations of: “Chronic Kidney Disease” OR “CKD” AND “screening” OR “awareness” AND “Nigerian Navy” OR “military health” OR “armed forces” AND “Nigeria”.

Reference lists of included articles were manually screened to identify additional relevant sources.

### Stage 3: Study Selection (Eligibility, Screening, and Selection Process)

Study selection followed the Population–Concept–Context (PCC) framework recommended for scoping reviews^13^.

The search yielded 889 records. After removal of 782 duplicates, 107 titles and abstracts were screened for relevance. Forty full-text articles were assessed for eligibility using the restricted PCC criteria. Thirty-two full-text articles were excluded for predefined reasons, and eight studies met the final inclusion criteria.

**Figure 1:**
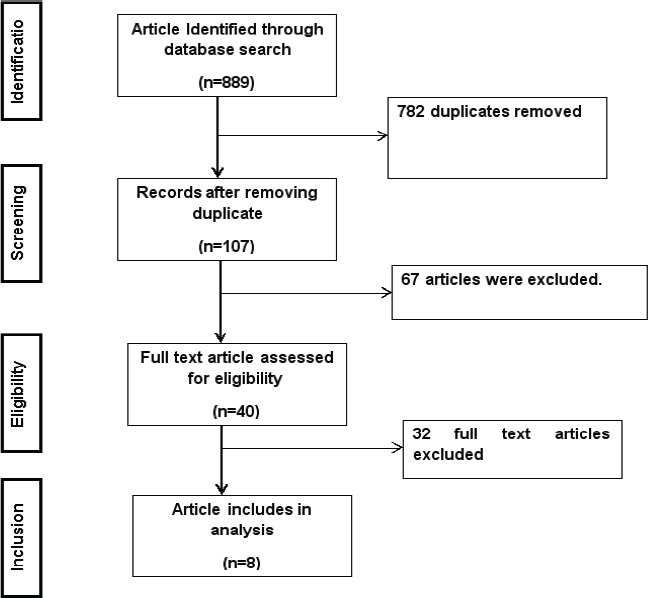
The PRISMA Framework for the Study

Screening and selection were conducted independently by two reviewers. Discrepancies were resolved through consensus, with arbitration by a third reviewer where necessary. The study selection process is illustrated in Figure 1 (PRISMA-ScR Flow Diagram).

### Stage 4: Charting the Data

Data were extracted using a standardized charting matrix capturing author, year, setting, study design, population, CKD domain, key findings, and relevance to Nigerian Navy health services. Extracted data were organized thematically^14^.

### Stage 5: Collating, Summarizing, and Reporting Results

A deductive thematic synthesis was applied across five predefined domains aligned with naval health service structure:

1. Epidemiology and registry systems
2. Screening and diagnostic infrastructure
3. Human-resource and training capacity
4. Dialysis and specialist care access
5. Governance and policy frameworks

Findings were synthesized narratively without meta-analysis, consistent with scoping review methodology.

## Data Management and Ethical Considerations

References were managed using Microsoft Excel 365 and Mendeley Reference Manager. Formal risk-of-bias appraisal was not undertaken, as it is not mandatory for scoping reviews. Ethical approval was not required because the study synthesized publicly available literature and institutional reports^15^. However, care was taken to acknowledge all data sources and to protect institutional confidentiality where internal Navy or Ministry of Defence documents were used.

### Justification for Timeframe (2000–2024)

This period reflects the maturation of modern CKD definitions (KDIGO) and aligns with major Nigerian Navy medical reforms initiated after the year 2000, improving comparability of diagnostic standards.

### Population

Active-duty Nigerian Navy personnel, reserve/retired naval personnel, and individuals receiving care specifically within Nigerian Navy Medical Services. Civilian populations, family dependents, and healthcare workers are excluded unless directly linked to CKD service delivery inside Navy facilities.

### Concept

Studies addressing chronic kidney disease awareness, prevention, screening, diagnostic capacity, workforce readiness, dialysis availability, and renal-related policy or governance relevant to the Nigerian Navy health system.

### Context

Nigerian Navy Medical Services (NNMS) and naval healthcare structures within Nigeria.

Military health systems in other LMICs are used only as benchmark comparators, not as included populations.

### Justification for Deductive Thematic Approach

A deductive approach to thematic analysis was applied because scoping reviews typically map evidence into predefined domains rooted in policy and service-delivery frameworks. The Nigerian Navy's CKD service continuum epidemiology, screening, workforce capacity, dialysis access, and governance guided theme development. This ensured structured synthesis aligned with the review objectives and the PCC framework.

### Characteristics of Included Studies

Figure 1. PRISMA-ScR Flow Diagram of Study Selection Records identified through database and grey-literature searches (n = 889)

Duplicates removed (n = 782)
Records screened by title and abstract (n = 107)
Full-text articles assessed for eligibility (n = 40)

Full-text articles excluded (n = 32), with reasons:

Not involving Nigerian Navy or military populations (n = 14)
Civilian-only CKD studies with no military relevance (n = 9)
No CKD-specific outcomes reported (n = 5)
Editorials, conference abstracts, or non-eligible publication types (n = 4)
Studies included in final synthesis (n = 8)

A total of eight studies met the final inclusion criteria after applying the restricted Population–Concept–Context (PCC) framework. These comprised four studies directly examining naval or military occupational risk environments, and four grey-literature documents from military health systems and Nigerian Navy Medical Services published between 2000 and 2024, including recent policy and surveillance reports. All included studies were directly relevant to Nigerian Navy personnel, naval healthcare structures, or comparable military operational environments, with no civilian-only studies retained. The PCC framework that guided the selection process is presented in Table 1, while Table 2 summarizes each included study's author, year, setting, design, CKD domain, key findings, relevance to naval health, and evidence type.

**Table 1:** PCC Inclusion and Exclusion Framework for Study Selection

Domain	Inclusion Criteria	Exclusion Criteria
**Population**	Active-duty or retired Nigerian Navy personnel; individuals receiving CKD-related care inside Nigerian Navy health facilities.	Dependents, civilian-only populations, and unrelated healthcare worker cohorts.
**Concept**	Studies addressing CKD awareness, knowledge, screening, diagnostic infrastructure, workforce, dialysis access, or policy/governance issues.	Studies unrelated to CKD; papers lacking empirical or programmatic renal data.
		Studies outside Nigeria with no military or occupational-health relevance.
**Context**	Nigerian Navy health system; comparable military health systems in Africa, Asia, or other LMICs.	Studies involving dependents, general base communities, or healthcare workers unless directly linked to CKD service provision for naval personnel.
**Study Design**	Cross-sectional, cohort, case-control, intervention, or policy/administrative reports published 2000 – Apr 2024 (English).	Editorials, letters, conference abstracts, or publications before 2000 or non-English.
**Outcomes**	CKD awareness levels, screening coverage, diagnostic capacity, nephrology workforce, dialysis services, and policy frameworks.	Articles without measurable outcomes on CKD awareness or service delivery.

**Table 2:** Summary of Included Studies (2000 – 2025)

S/N	Author (Year)	Country / Setting	Study Type	Population	CKD Domain	Key Findings	Relevance to Nigerian Navy	Evidence Type
1	Durotoye et al. (2019)	Nigeria – Shipyard Workers	Cross-sectional	Naval-affiliated shipyard workers	Occupational CKD risk; renal function	Heat exposure, solvents, and chemicals significantly impair renal function	Direct occupational relevance to Nigerian Navy engineering & marine personnel	Peer-reviewed
2	Souza et al. (2019)	Brazil – Navy Personnel	Occupational Study	Active-duty Brazilian Navy personnel	Dehydration & heat stress	Dehydration episodes and high heat index correlate with early renal impairment	Strong analogue for NN operations on ships and coastal environments	Peer-reviewed
3	Navy & Marine Corps Public Health Center (2020)	United States Navy	Military Medical Report	Active-duty US Navy personnel	Screening & surveillance	CKD metrics integrated into annual physical exams; renal registry operational	Benchmark for screening policy design in NN	Grey Literature
4	Indian Armed Forces Medical Services (2019–2021)	India – Armed Forces	Annual Health Report	Army/Navy/Airforce personnel	CKD monitoring & NCD control	Tele-nephrology, integrated registry, and structured annual renal checks	Comparative military best-practice for NN adoption	Grey Literature
5	Nigerian Navy Medical Services Reports (2021–2023)	Nigeria – NNMS	Administrative Reports	NN personnel & retirees	Dialysis services, laboratory capacity, workforce	NN dialysis centers exist only in Lagos; no CKD registry; no routine screening	Internal evidence of system gaps in NN	Grey Literature
6	Durotoye & Ajayi (2017)	Nigeria – Maritime workers	Cross-sectional	Marine workers with chemical/heat exposure	Occupational nephrotoxicity	Chronic occupational exposure increases CKD risk	Mirrors risks in naval dockyard/technical units	Peer-reviewed
7	Nigerian Federal Ministry of Health (NCD Policy 2019)	Nigeria	Policy document	National population (contextual)	NCD governance	No military-specific CKD strategy; naval adaptation required	Governance gap affecting NN	Grey Literature
8	WHO NCD Profiles (2022)	Sub-Saharan Africa	Global/National Evidence	African populations	Epidemiology	High CKD burden in SSA due to hypertension, diabetes, late diagnosis	Contextualizes national risk environment for NN personnel	Global Evidence

Following application of the corrected Population–Concept–Context (PCC) framework, only studies directly involving naval populations, maritime/military occupational exposures, or governance systems relevant to the Nigerian Navy were included in the final synthesis. Civilian-only CKD studies and foreign general-population studies were excluded because they did not meet the restricted population criteria required by the revised title and reviewer guidance. A separate subsection (“Comparator Evidence from Non-Naval Settings”) now contains non-military CKD studies used only to contextualize Nigerian Navy health risks.

### Epidemiology and Registry Systems

There is currently no dedicated renal registry within the Nigerian Navy Medical Services (NNMS). Available prevalence data are extrapolated from Nigerian community and hospital-based studies, which report CKD rates ranging from 11.4 % to 26 % depending on diagnostic criteria and population subgroup^16^,^17^. These findings suggest that a considerable number of naval personnel could have undiagnosed kidney disease, particularly among those with hypertension, diabetes, or long-term exposure to operational stressors. The absence of a formal renal database limits surveillance and planning. In contrast, the United States Navy and Indian Armed Forces maintain institutional renal registries integrated into annual health assessments^18^,^19^.

### Screening and Diagnostic Infrastructure

Routine screening for chronic kidney disease (CKD) through urinalysis, serum creatinine, and estimated glomerular filtration rate (eGFR) is not yet institutionalized across Nigerian Navy facilities. The Naval Reference Hospitals in Lagos and Calabar maintain well-equipped clinical chemistry laboratories with reliable reagent supplies for standard biochemical testing; however, they currently lack specialized diagnostic equipment such as automated microalbumin analyzers and eGFR-capable platforms required for advanced renal evaluation. Remote naval clinics and deployed medical detachments rarely have access to point-of-care devices for creatinine or eGFR, limiting early detection in field settings. Grey-literature sources from the NNMS (2021–2023) confirm that renal parameters are not routinely included in pre-deployment or periodic health assessments. This pattern reflects broader national challenges, where CKD screening remains opportunistic rather than preventive^20^,^21^.

### Human-Resource and Training Capacity

Nigeria has fewer than 250 practicing nephrologists, and only a limited number serve as visiting consultants to naval hospitals^22^. Most naval medical officers, nurses, and laboratory scientists have minimal training in renal medicine, and continuing professional-development programmes rarely include nephrology components. The shortage of renal nurses and dialysis technicians further limits the Navy's capacity for CKD management. A cross-sectional survey of nurses in South-West Nigeria found that only 6 % demonstrated adequate CKD knowledge^23^. This knowledge deficit highlights the need to integrate nephrology modules into military medical curricula and support staff in short-term renal fellowships or training with the Nigerian Association of Nephrology.

### Access to Dialysis and Specialist Care

Dialysis service provision within the Nigerian Navy has improved in recent years. The Naval Medical Centre, Victoria Island, and the Nigerian Navy Reference Hospital, Ojo (both in Lagos) currently operate functional dialysis units serving active-duty personnel, retirees, and dependents. Despite this progress, coverage remains limited to metropolitan areas. Personnel stationed outside Lagos continue to depend on civilian tertiary hospitals, such as University Teaching Hospitals near the naval bases where they are serving, for renal management / replacement therapy^24^. Delays in referral approvals and high out-of-pocket costs continue to compromise access and outcomes. Experiences from the United States Navy and Indian Armed Forces indicate that establishing additional in-house dialysis and tele-nephrology services can improve continuity of care and reduce treatment costs^18^,^19^. Expansion of dialysis capacity to the Naval Reference Hospital in Calabar and other NN Medical facilities in Sapele, Warri, and Port Harcourt, among others, is recommended.

### Governance and Policy Frameworks

The Nigerian Navy currently lacks a formal policy or strategic framework addressing CKD or broader non-communicable disease (NCD) management. The 2019 National NCD Policy provides general civilian guidelines but does not include adaptations for military or occupational-health contexts. Internal Navy health directives from 2020 to 2023 focus mainly on infectious diseases and general medical fitness but make little reference to CKD screening or renal-data systems^25^. By contrast, the U.S. Navy Bureau of Medicine and Surgery and the Indian Armed Forces Medical Services include CKD metrics within their integrated disease-surveillance platforms^18^,^19^. Institutionalizing CKD prevention within naval policy would align the Nigerian Navy with best practices in global military health systems and strengthen long-term operational readiness.

### Cross-Cutting Synthesis

Evidence across all thematic domains demonstrates fragmented CKD-prevention services, limited screening and diagnostic capacity, and underdeveloped nephrology infrastructure within the NN. Awareness among healthcare workers and personnel remains low, and routine renal-health assessments are not institutionalised. The two existing dialysis units in Lagos represent significant progress but remain insufficient for the Navy's nationwide operations. Addressing these service gaps through periodic screening, registry establishment, targeted workforce training, and policy integration would enhance both clinical care and mission preparedness.

## Discussion

This updated scoping review provides the first structured synthesis of chronic kidney disease (CKD) awareness, prevention, screening practices, and policy frameworks within the Nigerian Navy. By restricting the population to naval personnel and Navy medical facilities, the findings now offer a precise representation of CKD-related service gaps within the Navy's operational healthcare environment. Comparative insights from other military health systems are provided solely to benchmark potential improvements.

### Comparison with Civilian and Regional Evidence

The pattern observed in this review is consistent with reports from Nigeria's civilian population, where CKD prevalence has been estimated between 11% and 26%^26^,^27^. Similar studies across sub-Saharan Africa attribute this high burden to poorly controlled hypertension, diabetes, late presentation, and limited diagnostic capacity^28^. In the Nigerian Navy context, these civilian drivers are compounded by occupational and environmental exposures, including prolonged dehydration, physical stress, and periodic use of nephrotoxic agents aboard ships and in engineering workshops. Comparable findings were reported among naval personnel in Brazil and India, where operational factors were associated with higher risk of renal impairment^29^.

Unlike the United States Navy and Indian Armed Forces, which maintain renal registries and perform routine eGFR and urinalysis screening during annual physical examinations^30^,^31^, the Nigerian Navy lacks an integrated renal database or dedicated NCD surveillance platform. The absence of such systems impedes early detection and data-driven policy formulation. While the recent establishment of dialysis units at the Naval Medical Centre, Victoria Island, and the Nigerian Navy Reference Hospital, Ojo, represents a commendable milestone, these facilities remain geographically limited and insufficient for nationwide coverage.

### Workforce, Infrastructure, and Policy Gaps

Human-resource limitations remain a major barrier to CKD prevention and care within the Nigerian Navy. Nigeria currently has fewer than 250 practicing nephrologists and a limited number of trained renal nurses and technologists^32^. Within naval facilities, most healthcare providers have minimal exposure to nephrology or dialysis management. This contrasts with the Indian Armed Forces Medical Services, which operate structured nephrology rotations and postgraduate training for military physicians^31^. Expanding training for Nigerian Navy medical officers and laboratory scientists through partnerships with the Nigerian Association of Nephrology and accredited civilian institutions would substantially enhance in-house capacity.

From a policy standpoint, the absence of a Navy-specific CKD or non-communicable disease (NCD) framework reflects a broader institutional gap in occupational health governance. The 2019 National NCD Policy does not explicitly address military personnel or the peculiarities of maritime operations^33^. Incorporating CKD and other NCDs into the Navy's medical policy would improve long-term health planning and align with Sustainable Development Goal (SDG) target 3.4, which aims to reduce premature mortality from NCDs by one-third by 2030.

### Feasibility and Cost Implications

Implementing CKD screening within the Nigerian Navy is feasible using low-cost, high-yield strategies. Urine dipstick testing combined with serum creatinine estimation during recruitment, routine medicals, and pre-deployment evaluations could detect early renal impairment at minimal cost^34^. Point-of-care testing (POCT) devices for creatinine and eGFR can be deployed in base clinics and ships to enable on-site monitoring. A phased implementation approach is recommended;

**Phase I (0–6 months):** integrate dipstick urinalysis and serum creatinine testing into annual medical assessments.
**Phase II (6–18 months):** establish a centralized Navy CKD registry at the NN Medical Services Headquarters; train medical and laboratory personnel.
**Phase III (18–36 months):** expand tele-nephrology consultations, establish additional dialysis suites at Calabar, Port Harcourt, and Warri areas, and link the NN registry with national NCD databases.

Although cost estimates are context-dependent, studies in similar LMIC military settings indicate that early CKD screening is 8–10 times cheaper than dialysis initiation^35^. The adoption of POCT creatinine analysis, estimated at less than USD 2 per test, is therefore economically justifiable and sustainable.

### Operational Relevance and Readiness Implications

Untreated CKD among active-duty personnel undermines force readiness through reduced physical performance, fatigue, and increased risk of medical evacuation. Implementing a structured CKD prevention programme would improve long-term deployability and reduce healthcare costs. Routine renal assessments could also serve as a model for wider non-communicable-disease monitoring, thereby promoting a culture of preventive medicine across the Nigerian Navy. The operational relevance of renal health surveillance has been demonstrated in the United States Navy, where such systems have improved early intervention rates and reduced loss of man-hours due to chronic illness^30^.

## Strengths and Limitations

This review followed the PRISMA-ScR methodology to ensure transparency and reproducibility. The inclusion of both peer-reviewed and grey literature enriched the contextual understanding of CKD services within the Nigerian Navy. However, several limitations exist. First, the paucity of Navy-specific primary data restricted the depth of analysis, necessitating reliance on analogous civilian and foreign military sources. Second, the heterogeneity of included studies precluded quantitative synthesis or meta-analysis. Third, limited access to internal military health reports constrained verification of certain service-level claims. Nonetheless, the triangulated approach used here provides a credible overview of existing knowledge, gaps, and policy opportunities.

## Summary of Key Findings

CKD prevention, screening, and surveillance are not yet institutionalised within the Nigerian Navy health system. Awareness and training among healthcare personnel are low, mirroring civilian gaps.

Two functional dialysis units exist (Victoria Island and Ojo), but geographic access remains limited.

There is no renal registry, formal CKD policy, or integration with national NCD frameworks.

Cost-effective screening and tele-nephrology interventions are feasible and operationally beneficial.

## Conclusion

This scoping review reveals substantial gaps in chronic kidney disease (CKD) prevention, awareness, and policy implementation within the Nigerian Navy (NN). While the Service has made commendable progress through the establishment of two functional dialysis units, the Naval Medical Centre, Victoria Island, and the Nigerian Navy Reference Hospital, Ojo, these facilities remain confined to Lagos, leaving other major naval formations without access to specialized renal care. Laboratory services at reference hospitals such as Lagos and Calabar are generally well-stocked with reagents for clinical chemistry testing; however, the absence of specialized diagnostic equipment for advanced renal assessment, including automated eGFR analyzers and microalbuminuria testing platforms, limits the comprehensiveness of CKD diagnosis and monitoring.

The lack of a centralized renal registry, limited nephrology-trained workforce, and non-integration of CKD screening into annual health evaluations have collectively constrained the Navy's capacity for early detection and long-term renal health management. When compared to established systems in the United States and Indian armed forces, the Nigerian Navy's renal-care framework remains underdeveloped. Nonetheless, opportunities exist for reform through the deployment of low-cost, high-yield screening strategies, tele-nephrology integration, and policy mainstreaming.

Implementing CKD prevention and management strategies across naval medical commands will not only improve the health and welfare of personnel but also enhance operational readiness, reduce medical evacuation costs, and ensure alignment with national non-communicable disease (NCD) control objectives^36^,^37^.

## Recommendations

Given the clarified population scope and the refined evidence base, the following recommendations are specifically tailored to the Nigerian Navy Medical Services (NNMS). They address structural, clinical, operational, and policy gaps identified in the review and align with global best practices for military renal health management.

1. Integrate CKD Screening into Routine Naval Health Evaluations
  - Incorporate urinalysis and serum creatinine testing into recruitment, annual, and pre-deployment medical assessments.
  - Deploy portable point-of-care devices for creatinine and eGFR analysis in ships, base clinics, and field units [38].
2. Expand Renal Care Infrastructure Beyond Lagos
  - Establish additional dialysis and renal diagnostic centres at Naval Reference Hospitals Calabar, Warri, Port Harcourt and Sapele, which serve high-density operational commands.
  - These expansions would decentralize access, reduce travel-related delays, and strengthen emergency readiness for deployed personnel.
3. Strengthen Diagnostic and Laboratory Capacity
  - Ensure continuous reagent supply chains while acquiring specialized diagnostic equipment (e.g., microalbumin analyzers, nephrometry-capable autoanalyzers, and POCT systems).
  - Introduce standardized renal panels across Navy medical laboratories.
4. Enhance Human-Resource Development and Capacity Building
  - Integrate nephrology modules into continuing professional development (CPD) curricula for naval physicians, nurses, and laboratory scientists.
  - Sponsor postgraduate fellowships and short-term renal-care training programmes in collaboration with the Nigerian Association of Nephrology and tertiary hospitals.
5. Establish a Navy CKD and NCD Registry
  - Develop a digital renal registry within the NNMS to capture screening data, treatment outcomes, and follow-up indicators.
  - Link this registry with the national NCD surveillance system to facilitate coordinated health planning.
6. Adopt a Navy CKD Policy Framework
  - Formulate a service-specific CKD prevention and control policy that defines screening schedules, referral protocols, and budgetary responsibilities.
  - Embed CKD monitoring in the Navy's occupational health and wellness policy to ensure sustainability^39^.
7. Leverage Tele-Nephrology and Civil–Military Partnerships
  - Establish partnerships with civilian tertiary hospitals and private nephrology practices to provide remote specialist consultations, particularly for outstation commands.
  - Utilize existing Navy telemedicine infrastructure to support real-time management of CKD cases and second opinions^40^.
8. Sustain Research and Continuous Evaluation
  - Encourage operational research on CKD risk factors among naval personnel and dependents.
  - Periodically review implementation outcomes to guide continuous quality improvement and policy refinement.

## Summary Statement

The Nigerian Navy is well-positioned to integrate renal health into its broader non-communicable disease agenda. By expanding infrastructure to Naval Healthcare Facilities in Calabar, Warri, Port Harcourt, and Sapele, strengthening laboratory diagnostics, training personnel, and institutionalizing CKD surveillance, the Service can build a sustainable renal-care framework aligned with global military and national health priorities.

## References

[1] Jha V, Garcia-Garcia G, Iseki K, Li Z, Naicker S, Plattner B (2013). Chronic kidney disease: global dimension and perspectives. Lancet.

[2] Luyckx VA, Tonelli M, Stanifer JW (2018). The global burden of kidney disease and the sustainable development goals. Bull World Health Organ.

[3] Ulasi II, Ijoma CK (2010). The enormity of chronic kidney disease in Nigeria: the situation in a teaching hospital in South-East Nigeria. J Trop Med.

[4] Afolabi MO, Abioye-Kuteyi EA, Arogundade FA, Bello IS (2009). Prevalence of chronic kidney disease in a Nigerian family practice population. S Afr Fam Pract.

[5] Durotoye IA, Ajayi OE, Lawal MA (2019). Occupational hazards and renal function among shipyard workers in Lagos, Nigeria. Nig J Health Sci.

[6] Clark WF, Sontrop JM, Huang SH, Moist L, Bouby N, Bankir L (2016). Hydration and chronic kidney disease prevention: A review of evidence. Am J Nephrol.

[7] Bello AK, Levin A, Tonelli M, Okpechi IG, Feehally J, Harris D (2017). Global kidney health atlas: towards better kidney care. Kidney Int Suppl.

[8] Navy and Marine Corps Public Health Center (2020). Annual medical surveillance report.

[9] Ministry of Defence, Government of India (2020). Annual Report 2019–2020.

[10] Federal Ministry of Health (FMOH) (2019). National Policy and Strategic Plan of Action on Non-Communicable Diseases 2019.

[11] Arksey H, O'Malley L (2005). Scoping studies: towards a methodological framework. Int J Soc Res Methodol.

[12] Tricco AC, Lillie E, Zarin W, O'Brien KK, Colquhoun H, Levac D (2018). PRISMA extension for scoping reviews (PRISMA-ScR): checklist and explanation. Ann Intern Med.

[13] Levac D, Colquhoun H, O'Brien KK (2010). Scoping studies: advancing the methodology. Implement Sci.

[14] Peters MDJ, Marnie C, Tricco AC, Pollock D, Munn Z, Alexander L (2020). Updated methodological guidance for the conduct of scoping reviews. JBI Evid Synth.

[15] Arkell J, Rajapakse N, Delaney F, Khawaja Z (2022). Mapping kidney disease awareness and service availability in low- and middle-income countries: a scoping review. BMC Nephrol.

[16] Stanifer JW, Jing B, Tolan S, Helmke N, Mukerjee R, Naicker S (2014). The epidemiology of chronic kidney disease in sub-Saharan Africa: a systematic review and meta-analysis. Lancet Glob Health.

[17] Afolabi MO, Arogundade FA, Bello IS (2015). Prevalence and correlates of chronic kidney disease in Nigeria. Nephrol Dial Transplant.

[18] U.S. Navy Bureau of Medicine and Surgery (2020). Navy medical readiness report 2020.

[19] Indian Armed Forces Medical Services (AFMS) (2021). Annual health report 2021.

[20] Nigerian Navy Medical Services (NNMS) (2023). Annual Health and Logistics Report 2021–2023.

[21] Okwuonu CG, Chukwuonye II, Adejumo OA, Agaba EI (2014). Awareness level of chronic kidney disease in a rural community of South-East Nigeria. Niger J Clin Pract.

[22] Bello AK, Abioye-Kuteyi EA, Arogundade FA (2017). Chronic kidney disease: the Nigerian nephrology workforce crisis. Clin Kidney J.

[23] Adejumo O, Iyawe I, Afolayan A, Agaba E, Okoye J (2018). Nurses' knowledge of chronic kidney disease prevention and management in South-West Nigeria. J Ren Care.

[24] National Association of Nephrology (2023). Directory of dialysis facilities in Nigeria 2023.

[25] Federal Ministry of Health (FMOH) (2021). National Health Policy 2021.

[26] Okoye O, Ojeh-Oziegbe O, Oviasu E (2020). Epidemiology of chronic kidney disease in Nigeria: a systematic review. Niger J Clin Pract.

[27] Ulasi II, Chinwuba I, Ijoma CK, Onodugo OD, Arodiwe EB, Ifebunandu NA (2016). High prevalence and low awareness of CKD in a semi-urban community in South-East Nigeria. Nephrol Dial Transplant.

[28] Fabian J, Naicker S (2015). Chronic kidney disease in sub-Saharan Africa: an overview of determinants and management. Kidney Int Suppl.

[29] Souza AC, Oliveira M, Cunha L, Mendes M (2019). Renal function in Brazilian navy personnel exposed to heat and dehydration. Int J Occup Med Environ Health.

[30] U.S. Navy Bureau of Medicine and Surgery (2021). Operational medical surveillance manual.

[31] Indian Armed Forces Medical Services (2021). Comprehensive chronic disease management programme for service personnel.

[32] Okafor C, Okoye O, Okonkwo U, Nwankwo E (2022). Human-resource challenges in nephrology practice in Nigeria. Niger Postgrad Med J.

[33] World Health Organization (WHO) (2022). Noncommunicable diseases country profiles 2022.

[34] Moosa MR, Van der Walt I, Naicker S, Meyers AM (2015). Important causes of chronic kidney disease in South Africa: A review. S Afr Med J.

[35] Tannor EK, Archer E, Kapembwa K, van Schalkwyk C, Davids MR (2021). Cost-effectiveness of screening for CKD in low-income settings: experience from sub-Saharan Africa. Afr J Nephrol.

[36] Oladipo O, Omoniyi-Esan G, Akinsola W (2022). Policy and practice gaps in Nigeria's noncommunicable disease management. Afr Health Sci.

[37] United Nations Development Programme (UNDP) (2023). Sustainable Development Goal 3.4 Progress Report 2023.

[38] Johnson DW, Atai E, Chan M, Phoon RK, Scott C, Toussaint ND (2013). KHA-CARI guideline: early chronic kidney disease: detection, prevention, and management. Nephrology.

[39] Ministry of Defence, Nigeria (2022). Occupational Health and Safety Policy 2022.

[40] Bello AK, Levin A, Lunney M, Okpechi IG, Ye F, Osman MA (2023). Global Kidney Health Atlas 2023: pathways to improving kidney care. Kidney Int Suppl.

